# The THRIVE-CP trial - Targeted home-based training with real-time feedback to improve versatile movement behaviors and enhance outcomes in adolescents and young adults with Cerebral Palsy: Protocol for a randomized controlled trial

**DOI:** 10.1016/j.conctc.2025.101560

**Published:** 2025-10-15

**Authors:** Ivana Bardino Novosel, Jan Christensen, Mikkel Damgaard Justiniano, Astrid Siemens Lorenzen, Jakob Lorentzen

**Affiliations:** a Department of Neuroscience, Faculty of Health and Medical Sciences, The Panum Institute, Copenhagen University, Copenhagen, Denmark; b Department of Pediatric Neurology 5003, Copenhagen University Hospital, Rigshospitalet, Copenhagen, Denmark; c Department of Occupational Therapy and Physiotherapy, Copenhagen University Hospital, Rigshospitalet, Copenhagen, Denmark; d Department of Public Health, Section of Social Medicine, Copenhagen University, Copenhagen, Denmark; e The Elsass Foundation, Charlottenlund, Denmark; f Technical University of Denmark – DTU, Kongens Lyngby, Denmark

## 1 Background

Cerebral palsy (CP) is a permanent, non-progressive disorder caused by brain disturbances during fetal or infant development, affecting 1.5 to 3 out of every 1000 live births [1,2]. Symptoms vary widely, impacting functioning, body structures, activities, and societal participation, leading to disability. While CP is non-progressive, symptoms can evolve as individuals grow and societal expectations change. Multimorbidity is common among aging individuals with CP [3] possibly attributed to higher levels of sedentariness compared to peers [4], and a risk of decline in gross motor ability during adolescence [5]. This period may present an opportunity to intervene and prevent functional decline and health issues in adulthood.

Neuro(re-)habilitation utilizes the nervous system's inherent plasticity, promoting learning and adaptation through active participation, intensive training, task progression, goal setting, and motivation [[6], [7], [8]]. Improving and maintaining functional ability usually requires ongoing therapeutic engagement. However, limited resources often restrict access to supervised programs, particularly at high intensity. The necessity of traveling to a gym or clinic can further hinder attendance [9]. Home-based training shifts the emphasis from therapist-led sessions to self-initiated motor training. Evidence supports the feasibility and effectiveness of home-based training programs for infants and young children with CP, promoting integration into standard care [[10], [11], [12]]. These programs can improve balance [13], upper limb function, and parent satisfaction [14]. Significant improvements in upper limb skills were observed after eight weeks [14], while video-game-based training led to improvements across various functional tests after 20 weeks [15] with a reported average of 40 h of additional training. Compliance with home-based programs varies with self-reported adherence ranging from 56 % to 99 %. Recent findings suggest that extrinsic feedback from equipment or a coach enhances recovery and motor learning in stroke rehabilitation [16]. It's unclear if the effect arises from extrinsic feedback or is linked to higher intensity and adherence to training programs.

In the THRIVE-CP trial, we propose using the TIA app (Translating Intentions into Actions) and wearable Inertial Measurement Units (IMUs) for home-based motor training. The technology provides real-time performance feedback through music while objectively measuring attendance and adherence. Music Motion Feedback presents a potentially effective method to enhance meaningful changes in real-world movement behavior and functional improvement.

### 1.1 Objectives

The THRIVE-CP trial evaluates the effectiveness of incorporating Music Motion Feedback into home-based motor training for adolescents and young adults with CP compared to home-based motor training without Music Motion Feedback. We hypothesize that the Music Motion Feedback group will demonstrate greater improvements in real-world movement behavior, functional improvement, limb function, and perceived goal performance than the control group. Additionally, we expect the Music Motion Feedback group to attend and adhere more to the training program.

## 2 Methods

This protocol is reported according to the Standard Protocol Items: Recommendations for Interventional Trials (SPIRIT statement) [17].

### 2.1 Trial design

**Fig. 1:**
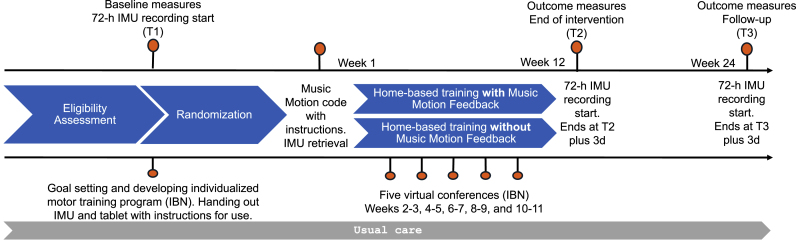
The THRIVE-CP trial design **Note:** Inertial Measurement Unit (IMU). Individualized motor training programs, tailored to individual goals, are created by IBN before randomization. These programs focus on three core components: 1) task specificity, 2) training intensity, and 3) virtual conferences. IBN hosts virtual conferences biweekly with each participant during the training period to improve adherence and adjust the program to optimize training intensity. A third-party allocator will visit participants after randomization, where the controls will receive a code that unlocks the TIA app on a tablet with the message “You are now ready for training.” In contrast, the Music Motion group participants will receive a code granting access to the Music Motion Feedback feature in the TIA app. Participants in both groups will engage in self-administered, home-based training for 12 weeks while wearing an IMU on the targeted extremity during training. Attendance and adherence data will be collected on the tablet for both groups, though not accessible to participants.

This is a two-arm parallel assignment randomized clinical superiority trial in which adolescents and young adults with CP self-administer home-based motor training with or without Music Motion Feedback. Outcomes will be assessed before randomization, after 12 weeks of intervention (end of intervention - T2), and to evaluate the sustainability of potential improvements after 24 weeks (follow-up - T3). The trial design is visually presented in Fig. 1.

### 2.2 Setting, trial site, and recruitment

Participants will be recruited consecutively from the CP Youth Clinic at Copenhagen University Hospital — Rigshospitalet, Denmark. The clinic's nurse and physical therapist will invite eligible individuals to participate during routine consultations (i.e., when individuals are 15, 17, and 21 years old). Individuals who have previously attended the CP Youth Clinic and consented to research invitations will be invited to participate.

### 2.3 Eligibility criteria

Individuals with CP aged 15 to 25 at enrollment are eligible to participate if they have sensorimotor deficits in at least one limb and are classified within GMFCS-E&R levels I-V. They must show motivation to develop new motor skills or recover lost ones, as indicated by their or their proxy's ability to express a specific goal they wish to achieve. They must be able to follow instructions independently or with caregiver support and be capable of providing informed consent to participate. Individuals will be excluded from participation if they have dyskinetic CP, significant health risks, have undergone major orthopedic or neurosurgical procedures within six months preceding enrollment or planned during the intervention period, or minor procedures that could temporarily affect training or movement behavior within one month before enrollment or during the trial. Eligibility for borderline cases is determined by the principal investigator in consultation with the treating physician. Botulinum neurotoxin type A (BoNT-A)-naïve individuals will be excluded if they have received intramuscular injections within three months before baseline. Individuals undergoing routine BoNT-A injections are eligible, and injections in muscles not directly targeted by the training program do not preclude participation.

### 2.4 Individualized motor training programs

Participants in both groups will engage in home-based motor training grounded in principles of neural plasticity [[6], [7], [8]]. After informed consent, an experienced physical therapist (IBN) will develop an individualized motor training program with each participant. The process includes setting a long-term goal using the Canadian Occupational Performance Measure (described under tertiary outcomes), conducting neurological, biomechanical, and motor control assessments to identify specific movement limitations, and focusing on key factors for goal achievement. Short-term goals will be established following the SMART framework [18]. Participants will wear a small wireless IMU (MetaMotions from Mbientlab, San Francisco, United States) on the targeted body part during training (e.g., on the thigh for sit-to-stand), which will transmit movement signals via Bluetooth to a tablet's TIA app. IMU and tablet will be provided.

**Task-specific training** emphasizes meaningful, everyday activities, emphasizing neural plasticity's dependence on experience. Addressing each participant's unique challenges and goals is essential. Programs will foster intentional, active practice and minimize reliance on verbal or physical assistance, using visual cues to guide movement.

**Intensity** emphasizes high effort and challenging training to induce neuroplastic changes. It will be approached as a dynamic interplay between frequency, repetition, difficulty, resistance, session duration, and overall program length, recognizing that optimal learning occurs under conditions that do not yield peak performance [19]. The training setups will allow participants to perform independently and at their convenience throughout the day.

**Virtual conferences** will be conducted by licensed physical therapists trained in the THRIVE-CP protocol. Five sessions of approximately 30 min each will promote adherence to the program and optimize training intensity. Participants' skill acquisition, training tolerance, and short-term goals will be reviewed to either advance progression or address regression while maintaining a balance between task difficulty and performance.

### 2.5 Music Motion feedback

The Music Motion Feedback group will use a specific code to access the feedback feature in the TIA app for their motor training program. This app provides real-time auditory feedback to inform participants if they fail to meet the required intensity during practice.

TIA converts signals (from accelerometers and gyroscopes) gathered from IMUs worn on targeted body parts into commands. Originally designed for users with movement disorders to control devices like music volume and lights, the app has been adapted to support a rehabilitative approach, where music becomes distorted if the participant does not meet the training intensity threshold.

Before each session, the TIA app guides participants through a process to set their intensity thresholds. This involves participants moving as quickly and precisely as possible through the training movements while utilizing their full range of motion. Each participant will have an individual percentage threshold in their training program based on factors such as prescribed training frequency, repetitions, difficulty, resistance, session duration, and the overall length of the program. During training, each complete cycle of movement that reaches the intensity threshold (as a percentage of the current maximum) will trigger specific musical elements like a drumbeat or vocals in the participant's chosen music. If the participant fails to meet the threshold, elements of the music will drop out, providing error-related auditory feedback to encourage correction.

### 2.6 Randomization

Participants will be assigned to groups using matched pairs randomization with a 1:1 allocation ratio. Matching will be based on dichotomized ages (adolescents 15–19 years, young adults 20–25 years) and GMFCS-E&R classifications. Within each stratum, participants will be grouped into pairs. A computer-generated randomization sequence will assign the first participant in each pair to either group, with the second assigned to the alternate. IBN will enroll participants and extract age and GMFCS-E&R classifications to create pairs. A third-party allocator will conduct randomization to ensure allocation concealment, with the sequence securely stored and revealed only after statistical analysis is complete.

### 2.7 Blinding

Outcome assessors, the physical therapist responsible for developing the motor training programs and conducting virtual conferences (IBN), participants, and the statistician will be blinded to group allocation. To minimize expectation bias participants will not be informed that only one group will receive Music Motion Feedback during training. Participants will be instructed not to discuss or disclose their allocation with outcome evaluators. Virtual conferences will focus exclusively on motor training programs and goals, deliberately omitting any reference to intervention features like Music Motion Feedback.

### 2.8 Concomitant care and interventions

All participants should maintain their usual care routines. This care may include, but is not limited to, services provided by physical therapists, occupational therapists, speech therapists, and medical professionals in managing spasticity, pain, or seizures [1].

### 2.9 Criteria for modifying or discontinuing interventions and participation

Changes to the motor training program may occur outside of the standard progression or regression, provided the goals are maintained. These occur during scheduled virtual conferences, either at the participants' request, in response to reported musculoskeletal issues, or due to safety concerns. If a participant requires surgery affecting functional ability and training during the trial, faces an illness that necessitates more than a week of hospitalization, or if Botox-naïve participants receive injections, their participation will be discontinued. If a participant withdraws from the trial, discontinues the motor training program, or is lost to follow-up, they will be asked whether the collected data can be used for analysis.

### 2.10 Outcomes

**Fig. 2:**
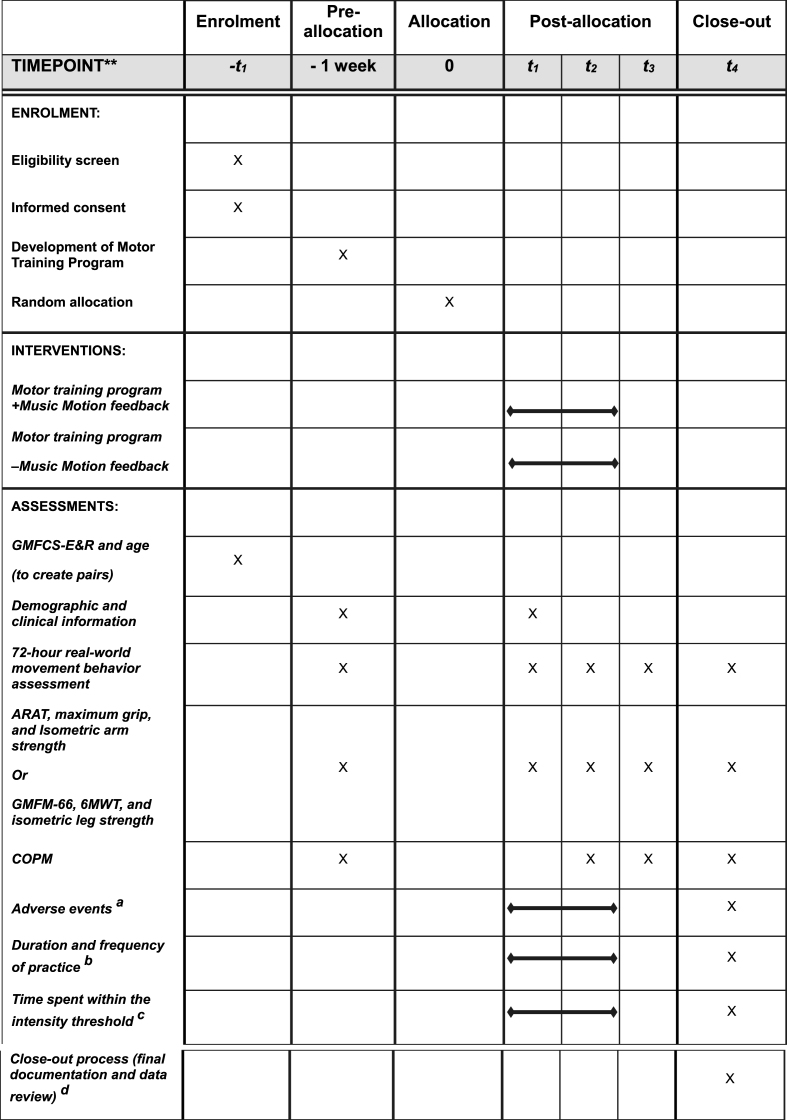
SPIRIT figure. Schedule of enrollment, interventions, and assessments. **Note:***t*_1_ baseline, *t*_2_ 12 weeks (primary endpoint), *t*_3_ 24 weeks, *t*_4_ 26 weeks. Gross Motor Functional Classification Scale-Expanded and Revised (GMFCS-E&R), Action Research Arm Test (ARAT), Gross Motor Function Measure-66 (GMFM-66), 6-min walk test (6MWT), Canadian Occupational Performance Measure (COPM). (a) Systematic inquiry of potential events during virtual conferences. (b) Data will be collected continuously through the TIA app. (c) Only the Music Motion feedback group can be tracked for staying at or above the intensity threshold. (d) Involves completing the participant's involvement in the trial, including final data review, administrative tasks, and confirming that all study procedures have been appropriately completed.

Baseline (T1) and outcome assessments (T2, T3) will be performed on weekdays within a week before intervention commencement (T1), within a week after the end of intervention (T2), and 24 weeks after intervention initiation (T3). Assessments will be conducted at approximately the same time of day for each participant to minimize intraday variability. The primary endpoint is T2. Please view the assessment timeline in Fig. 2.

#### 2.10.1 Primary outcome

The primary outcome is the percentage change in real-world daytime movement of the targeted extremity (right or left arm or leg). We hypothesize this reflects the impact of the anticipated increase in capacity from motor training programs on real-world movement behaviors. Data will be collected from seven IMUs worn by participants for 72 consecutive hours and analyzed using a deep-learning neural network. The methodology details have been previously described [20]. IMUs will be attached with adhesive patches on the sternum, wrists, thighs, and lower legs to collect 3-axis accelerometer and gyroscope data. A custom neural network converts the signals into images and processes them through several convolutional layers, extracting features to recognize movement behaviors. Movement behaviors beyond limb movement will be utilized for exploratory outcomes. Daytime will be recorded in participant logs, noting the times in bed, lights out, devices off, wakefulness, and when they leave bed.

#### 2.10.2 Secondary outcomes

The secondary outcomes will evaluate changes in capacity and function. Participants will be split based on goal-directed training for upper or lower extremities. The effects of upper-extremity training will be assessed using the Action Research Arm Test (ARAT) total score, isometric arm strength, and maximum grip strength. For lower-extremity assessment, the Gross Motor Function Measure-66 (GMFM-66), isometric leg strength, and the 6-min walk test will be applied. In addition, attendance and adherence will be monitored as process measures.

**ARAT** consists of 19 items across four dimensions: grasp, grip, pinch, and gross arm movement. Each item is rated on a 4-point ordinal scale ranging from normal performance to inability to perform any part of the test. The maximum total score is 57 points, with higher scores indicating greater functioning [21].

A hand dynamometer will determine the percentage change in **maximum hand grip strength** (Jamar Technologies, Horsham, PA, USA). Participants will squeeze the device maximally. After one practice trial, three measurements will be taken, with rest periods. The highest value will be recorded as the maximum grip strength.

**GMFM-66** is a 66-item observational assessment divided into five dimensions: Lying and rolling, sitting, crawling and kneeling, standing, walking, running, and jumping. Each performance is rated on a 4-point ordinal scale: Does not initiate task, Initiates task (<10 %), Partially completes task (10–99 %), and Completes task (100 %). Calculations of GMFM-66 scores require the use of a computer program, Gross Motor Ability Estimator-3 (GMAE-3) [22].

**6MWT** measures submaximal aerobic and functional gait capacity. Able participants will walk 6 min on a 30-m walkway with the enclosed corridor length marked at every 3-m interval. Cones mark turnaround points. The total distance walked over the 6 min is used as the score [23].

**Isometric strength** will be assessed using handheld dynamometry by measuring maximal isometric force for muscle groups. Three measurements will be recorded with rest periods, and the highest value (kilogram force) per muscle group will be recorded as the maximum.

The TIA app will collect **attendance and adherence data**, which includes daily logins, session duration, and the time spent moving the targeted extremity.

For participants in the Music Motion Feedback group, daily intensity thresholds and time spent within these thresholds will also be recorded and descriptively reported.

#### 2.10.3 Tertiary outcome

**The Canadian Occupational Performance Measure (COPM)** assesses perceived performance in activities of daily living, functional mobility, life participation, and occupational performance. Through a semi-structured interview, participants identify activities they want to, need to, or are expected to perform. Participants establish one long-term goal and rate their performance and satisfaction towards the set goal on a numeric ranking scale from zero to ten. A caregiver or proxy may respond on behalf of the participant.

#### 2.10.4 Exploratory outcomes

Three days (72 h) of movement behavior will be assessed with IMUs and analyzed using a deep-learning neural network [20]. We will describe the overall change in movement behavior patterns after the intervention, including changes in the time spent walking, standing, sitting, and lying down and the number of transitions between these states. We will also examine relative extremity usage.

### 2.11 Other trial data

**Adverse events** during the intervention period (T1-T2) will include both serious (e.g. death, hospitalization) and non-serious adverse events (e.g. pain, fatigue) [24]. Harms will be evaluated using relative risk between the Music Motion Feedback and control groups for both event types.

**Demographic data**, collected during baseline assessment through self- or proxy reports for all participants, will include age, sex, daytime and nighttime occurrence of pain, living arrangements, occupation status, and leisure time activities.

**Clinical information** such as GMFCS-E&R classification, type of CP, and affected body parts will be examined on-site during baseline assessment.

**Routine BoNT-A injections** (date and muscles) will be recorded based on participant or caregiver reports at the virtual conference and verified in medical records.

### 2.12 Data management

All data collection and handling will comply with data protection regulations to ensure participant confidentiality and privacy. Clinical assessments will be recorded on paper and uploaded for storage in a RedCap database.

### 2.13 Statistical analysis

A statistical analysis plan will be developed in collaboration with a biostatistician and uploaded to ClinicalTrials.gov before the last participant's first visit.

#### 2.13.1 Superiority margin

The change in real-world daytime extremity movement over 72 h from baseline to follow-up (T2) and long-term follow-up (T3) determines the superiority margin, set at a 10 % increase for both upper and lower extremities.

Due to the wide variability in extremity movement among adolescents and young adults with CP at GMFCS-E&R levels I-V (upper: 5.6 %–54.4 %; lower: 1.3 % - 19.4 % of daytime) (Novosel, manuscript), percentage change was chosen over absolute change. Superiority is claimed if the lower bound of the 95 % confidence interval for the Music Motion Feedback group surpasses the 10 % margin. While no universal threshold for clinically meaningful improvement exists, this margin aligns with chronic stroke findings, where a change of 575–752 accelerometer counts is considered a meaningful improvement (15 %-20 %) [25].

#### 2.13.2 Sample size

The sample size calculation is based on a systematic review and meta-analysis of controlled trials assessing extrinsic feedback on recovery and motor learning in individuals with brain injuries. The analysis found a significant Standardized Mean Difference of 0.76 with participants in identical motor training programs, differing only in feedback provision [16]. With 58 participants, we will attain a statistical power of 81.8 %. To account for potential dropouts, we plan to enroll 70 participants (35 in each group).

#### 2.13.3 Statistical methods

The distributions of continuous data will be assessed using Quantile-Quantile (Q-Q) plots, histograms, and the Shapiro-Wilk test. Normally distributed continuous data will be analyzed with parametric statistics. If normality is not met, a logarithmic transformation will be applied. Ordinal data will use non-parametric statistics. Categorical data will be presented as frequencies and percentages. An intention-to-treat analysis will include data from all trial participants.

##### 2.13.3.1 The primary outcome

A linear mixed-effects model (LMM) will analyze the percentage change in real-world targeted extremity movement from baseline (T1) to 12 weeks (T2) and 24 weeks (T3). The model will include fixed effects for Group (Music Motion Feedback vs. Control), Time (T1-T2, T1-T3), and their interaction (Group × Time), adjusted for baseline extremity movement time and sex. A random intercept for each participant will account for individual differences in baseline differences, and a random slope for Time will model variability in improvement rates. If residuals indicate non-normality, a logarithmic transformation will be performed.

Given the moderate sample size, the analysis will be conducted using restricted maximum likelihood estimation. A Holm-Bonferroni correction will be applied to adjust for multiple comparisons. A planned subgroup analysis will evaluate age group effects (adolescents vs. young adults).

##### 2.13.3.2 Secondary outcomes

Attendance (number of training sessions) and adherence (active practice time) will each be categorized based on the prescribed dose achieved: low (<33 %), medium (33–66 %), and high (>66 %). Chi-square tests will compare the two groups' attendance distribution and adherence levels.

The analysis of other secondary outcomes will follow the same approach as the primary outcome, using LMM.

##### 2.13.3.3 Tertiary outcomes

The Mann-Whitney *U* test will be employed to compare Canadian Occupational Performance Measure score changes between the two groups.

#### 2.13.4 Missing data

To address dropouts and discontinuations, including participants who meet exclusion criteria during the trial, we will use multiple imputations to generate overall estimates and standard errors that account for the uncertainty associated with the imputation.

#### 2.13.5 Sensitivity analysis

As an example, sensitivity analysis will evaluate whether the number of days since the most recent BoNT-A injection in targeted muscles affects the estimated treatment effect. For each assessment (T1, T2, T3), the days since the most recent BoNT-A injection in targeted muscles will be calculated and included as a covariate in the primary mixed-effects model. Additional sensitivity analyses will be pre-specified in the complete statistical analysis plan, which will be finalized and uploaded to ClinicalTrials.gov before the last participant's first visit.

### 2.14 Patient and Public Involvement

Patient and Public Involvement (PPI) was integrated into the THRIVE-CP trial design to ensure it aligns with the needs and perspectives of the target population. ASL, a young woman with lived experience of CP, contributed to identifying priority research questions and refining the protocol.

### 2.15 Ethics

The project will be approved by the Danish Health Research Ethics Committee before recruitment and will adhere to the Declaration of Helsinki [26]. Written informed consent will be obtained from eligible individuals, emphasizing voluntary participation and the right to withdraw without consequences. Parental permission will be sought for individuals aged 15 to 17. Age-appropriate information will be provided to ensure understanding of the implications of participation. Since the interventions are tailored to individual needs, it is not feasible to provide specific details about them before enrollment, as confirmed by the ethics committee (Case No. G-25025924).

### 2.16 Protocol amendments

Any protocol or statistical analysis plan amendments will be declared with reasons on ClinicalTrials.gov.

### 2.17 Dissemination of results

Findings will be shared to inform clinical practice and future research regardless of outcomes. Results will be published in peer-reviewed journals and presented at conferences. Outreach will involve the CP Youth Clinic at Rigshospitalet, with potential collaboration with the National Association for Cerebral Palsy Denmark. We will prioritize accessible communication through PPI involvement. Updates will be provided to the trial funder; however, they will not affect the design, analysis, interpretation, or reporting.

## 3 Discussion

Music Motion Feedback is a promising approach to improving movement behavior and functional improvement in adolescents and young adults with CP, potentially increasing activity and reducing disability. By providing real-time extrinsic feedback in tailored home-based training, we utilize established motor learning principles to enhance adherence and long-term outcomes. However, the THRIVE-CP trial faces generalizability limitations, such as recruiting intrinsically motivated participants and the bias of CP severity affecting participation, with 70 % of participants projected at GMFCS-E&R I-II. Additionally, those with lower technological literacy may struggle with adherence to the Music Motion Feedback. The trial lacks a usual care group due to recruitment constraints, limiting our ability to assess the separate effect of Music Motion Feedback. However, the TRIVE-CP trial addresses a critical challenge faced by adolescents and young adults with CP. If effective, this technology could provide an accessible and scalable method to enhance adherence to home-based motor training and foster meaningful progress in real-world movement behavior and functional abilities. The findings may significantly impact practice by promoting sustainable behavioral changes and improving the quality of life for individuals with CP.

## CRediT authorship contribution statement

**Ivana Bardino Novosel:** Writing – original draft, Methodology, Conceptualization. **Jan Christensen:** Writing – review & editing, Methodology. **Mikkel Damgaard Justiniano:** Writing – review & editing, Methodology. **Astrid Siemens Lorenzen:** Writing – review & editing, Methodology. **Jakob Lorentzen:** Writing – review & editing, Methodology, Funding acquisition, Conceptualization.

## Trial registration

ClinicalTrials.gov, Version 1, NCT06962618, Registered May 2025.

## Funding

The protocol is funded by the 10.13039/501100008340Elsass Foundation grant 21-B01-1474.

## Declaration of competing interest

The authors declare that they have no known competing financial interests or personal relationships that could have appeared to influence the work reported in this paper.

## Data Availability

No data was used for the research described in the article.
